# Association between the preoperative geriatric nutritional risk index and 90-day complications after open radical cystectomy: a retrospective cohort study

**DOI:** 10.3389/fsurg.2026.1987253

**Published:** 2026-10-29

**Authors:** Fatih Sandikci, Muhammed Yusuf Aydin, Mehmet Altan, Görkem Özenç, Özden Kokurcan, Berk Yasin Ekenci

**Affiliations:** 1 Department of Urology, Health Ministry of Türkiye, Ankara Etlik City Hospital, Ankara, Türkiye; 2 Department of Urology, Health Ministry of Türkiye, Etimesgut State Hospital, Ankara, Türkiye; 3 Department of Biochemistry, Health Ministry of Türkiye, Eskişehir Sivrihisar State Hospital, Eskişehir, Türkiye; 4 Department of Urology, Bayındır Hospital, Ankara, Türkiye

**Keywords:** bladder cancer, eras, geriatric nutritional risk index, ileal conduit, postoperative complications, radical cystectomy

## Abstract

**Background/objectives:**

Muscle-invasive bladder cancer requiring radical cystectomy is associated with substantial postoperative morbidity, particularly in elderly patients. This study evaluated the predictive capacity of the preoperative Geriatric Nutritional Risk Index (GNRI) for 90-day complications following open radical cystectomy with ileal conduit urinary diversion.

**Methods:**

We conducted a single-center retrospective cohort study of 80 consecutive patients who underwent open radical cystectomy and exclusively ileal conduit urinary diversion (100.0%) between November 2022 and December 2025. Preoperative GNRI was calculated from serum albumin and body weight metrics. Multivariable analysis was conducted using a 3-covariate Firth's penalized logistic regression model. An empirical threshold (GNRI < 94.5) derived from ROC analysis was evaluated alongside continuous GNRI scores.

**Results:**

The overall 90-day complication rate was 82.5% (66/80). Mean preoperative GNRI was 94.81 ± 7.24. The ROC-derived optimal cutoff for overall complications was GNRI < 94.5 (AUC = 0.628, Sensitivity: 71.2%, Specificity: 57.1%). While continuous GNRI was not an independent predictor of total complications in multivariable analysis (OR: 1.052 per 1-unit decrease, 95% CI: 0.948–1.168, *p* = 0.342), patients with GNRI < 94.5 exhibited significantly higher rates of specific severe morbidity subtypes, including infectious complications (67.7% vs. 34.7%, OR: 3.95, *p* = 0.004), gastrointestinal complications (74.2% vs. 36.7%, OR: 4.95, *p* = 0.001), and cardiovascular adverse events (25.8% vs. 4.1%, OR: 8.17, *p* = 0.005). Advanced pathological stage (≥pT3) remained the sole independent predictor of overall 90-day morbidity (OR: 4.187, *p* = 0.024).

**Conclusions:**

Preoperative GNRI is not an independent predictor of overall 90-day post-cystectomy complications; however, lower GNRI values (<94.5) strongly correlate with organ-specific infectious, gastrointestinal, and cardiovascular morbidity. GNRI serves as a valuable, objective screening tool within Enhanced Recovery After Surgery (ERAS) pathways to trigger targeted perioperative prehabilitation and nutritional optimization.

## 1 Introduction

Bladder cancer is the tenth most frequently diagnosed malignancy worldwide, with muscle-invasive bladder cancer (MIBC) representing a high-risk entity requiring aggressive multimodal treatment. Radical cystectomy with bilateral pelvic lymph node dissection remains the gold standard surgical intervention for non-metastatic MIBC and high-risk non-muscle-invasive bladder cancer refractory to intravesical therapies (1, 2). Despite substantial refinements in surgical techniques, anesthesia protocols, and perioperative care pathways, radical cystectomy continues to carry one of the highest morbidity rates among urological procedures, with 90-day postoperative complication rates ranging between 50% and 80% (3, 4).

The physiological burden of major extirpative surgery coupled with urinary diversion is particularly pronounced in geriatric patients, who frequently present with age-related functional decline, multiple chronic comorbidity burdens, and baseline nutritional depletion (5). Preoperative malnutrition is an established, independent risk factor for delayed wound healing, impaired immune competence, prolonged hospital length of stay, surgical site infections, and increased perioperative mortality across surgical oncology disciplines (6, 7). Consequently, accurate preoperative risk stratification and objective identification of vulnerable surgical candidates are paramount for optimizing clinical decision-making and implementing prehabilitation interventions.

The Geriatric Nutritional Risk Index (GNRI), originally formulated by Bouillanne et al. in 2005 for hospitalized elderly patients, is an objective, reproducible scoring system calculated from baseline serum albumin levels and the ratio of current to ideal body weight (8). While traditional nutritional assessments rely on subjective questionnaires or complex anthropometric measurements, GNRI offers a rapid, cost-effective screening metric. Previous studies in robotic radical cystectomy cohorts and general geriatric trauma populations have suggested that lower GNRI scores correlate with increased adverse surgical outcomes (9, 10). However, data regarding the prognostic utility of GNRI specifically in open radical cystectomy which incurs greater tissue trauma, surgical stress, and fluid shifts than minimally invasive approaches remain limited and conflicting.

Therefore, the primary objective of this study was to evaluate whether preoperative GNRI, assessed both as a continuous variable and via population-tailored cutoffs, serves as an independent predictor of 90-day postoperative complications in a homogenous cohort of patients undergoing open radical cystectomy with ileal conduit urinary diversion.

## 2 Materials and methods

### 2.1 Study design and ethical approval

This single-center retrospective cohort study was conducted at the Department of Urology, Ankara Etlik City Hospital, Ankara, Turkiye. Institutional review board and ethics committee approval was formally granted prior to study initiation (Ankara Etlik City Hospital Ethics Committee, Protocol Code: AEŞH-BADEK2-2026-111, Approval Date: February 10, 2026). All procedures performed were in strict compliance with the ethical standards of the 1964 Declaration of Helsinki and its later amendments. Due to the retrospective observational nature of the study using anonymized clinical registry data, the ethics committee waived the requirement for individual written informed consent.

### 2.2 Inclusion and exclusion criteria

We retrospectively screened consecutive adult patients (aged ≥18 years) who underwent open radical cystectomy with regional pelvic lymph node dissection for pathologically confirmed muscle-invasive bladder cancer or high-risk non-muscle-invasive bladder cancer between November 20, 2022, and December 1, 2025. To eliminate confounding variables associated with urinary diversion techniques, inclusion was strictly restricted to patients receiving ileal conduit urinary diversion (100.0%, *n* = 80/80). No patients undergoing orthotopic neobladder, continent cutaneous diversion, or ureterocutaneostomy were included.

Exclusion criteria were: incomplete medical, laboratory, or surgical records (e.g., missing baseline preoperative serum albumin); minimally invasive surgical approaches (laparoscopic or robotic radical cystectomy); emergency surgical procedures; palliative cystectomy for metastatic disease; and loss to follow-up prior to the 90-day postoperative landmark. Out of 84 patients initially screened, 4 were excluded due to missing preoperative laboratory records or early loss to follow-up, yielding a final analytical cohort of 80 patients who completed full 90-day follow-up by March 1, 2026.

### 2.3 Sample size and event-per-variable constraints

An *a priori* sample size calculation was conducted using G*Power software (v3.1.9.7) applying the “Exact – Logistic Regression” model based on anticipated effect sizes. Furthermore, to evaluate model stability and guard against model overfitting given our final sample size (*N* = 80) and outcome distribution (14 non-events vs. 66 events), we implemented Riley's pmsampsize package in R (11). Based on an outcome proportion of 0.825 and an anticipated Nagelkerke R² of 0.15, sample size guidelines indicated that having 14 non-events strictly limits multivariable modeling according to the Events-Per-Variable (EPV) principle. Consequently, our multivariable logistic regression model was strictly capped at a maximum of three predictor variables.

### 2.4 Data collection and GNRI calculation

Demographic characteristics (age, gender, BMI, smoking history), baseline comorbidity indices [Charlson Comorbidity Index [CCI], American Society of Anesthesiologists [ASA] physical status score], preoperative laboratory parameters [serum albumin, C-reactive protein [CRP], complete blood count metrics including Neutrophil-to-Lymphocyte Ratio [NLR], hemoglobin, eGFR], surgical metrics (operation time, intraoperative blood loss, blood transfusion requirement), and pathological staging data were systematically extracted from electronic medical records.

Preoperative serum albumin was measured within 7 days prior to surgery. Body mass index (BMI) was calculated as weight in kilograms divided by height in meters squared (kg/m²). Preoperative GNRI was calculated using the standardized formula established by Bouillanne et al. (8):

GNRI=(1.489×serumalbumin[g/L])+(41.7×currentbodyweight/idealbodyweight)

Ideal body weight was determined using the Lorentz formula based on height and gender. When current body weight exceeded ideal weight, the weight ratio was set to 1.0, as per convention (8).

In the original geriatric literature by Bouillanne et al. (8), GNRI values are categorized into four nutritional risk strata: severe risk (<82), moderate risk (82–92), low risk (92–98), and no risk (≥98). In surgical oncology studies (e.g., Yamada et al., Takahashi et al.), 92 serves as the cutoff for moderate-to-severe nutritional risk, while 98 marks the threshold for normal nutrition (9, 10). To thoroughly evaluate these historical principles, we examined both established cutoffs (<92 and <98). Furthermore, to prevent information loss caused by arbitrary categorization of continuous biological variables, GNRI was primarily analyzed as a continuous variable in logistic regression models. Additionally, Receiver Operating Characteristic (ROC) curve analysis was performed to derive an empirical, population-tailored cutoff for overall 90-day complications using the Youden index (J = Sensitivity + Specificity—1), yielding an optimal cohort threshold of GNRI < 94.5.

### 2.5 Outcome assessment and postoperative follow-up

The primary outcome was the occurrence of 90-day postoperative complications, categorized and graded according to the standardized Clavien-Dindo (CD) classification system. Complications were stratified into minor (CD Grades I–II) and major (CD Grades III–IV), while 90-day postoperative mortality was designated as CD Grade V (12). Specific complication subtypes including infectious (sepsis, surgical site infections, pyelonephritis), gastrointestinal (paralytic ileus, mechanical bowel obstruction, anastomotic leak), cardiovascular (myocardial infarction, arrhythmia, deep vein thrombosis/pulmonary embolism), wound healing deficiencies, and urinary complications were documented.

### 2.6 Statistical analysis

Statistical analyses were executed using SPSS Statistics version 25.0 (IBM Corp., Armonk, NY, USA) and R software (version 4.3.1). Continuous variables, including age, preoperative serum albumin, body mass index, C-reactive protein, neutrophil-to-lymphocyte ratio, and hemoglobin, were expressed as mean ± standard deviation or median (interquartile range) based on normality testing via the Shapiro–Wilk test. Group comparisons between Low GNRI (< 94.5) and High GNRI (≥ 94.5) cohorts for baseline demographic, continuous laboratory parameters (including serum albumin, BMI, CRP, NLR, hemoglobin, and eGFR), and categorical clinical variables were performed using Student's t-test or Mann–Whitney U test for continuous variables based on normality testing via Shapiro–Wilk test, and Chi-square or Fisher's exact test for categorical variables. The statistical layout of Table 1 was aligned with Table 2, providing two-sided *p*-values across all continuous and categorical parameters evaluated. Categorical variables were expressed as absolute frequencies and percentages (n, %). Differences between groups were analyzed using Student's t-test or Mann–Whitney U test for continuous data, and Chi-square test or Fisher's exact test for categorical variables. Univariable and multivariable logistic regression analyses were conducted. Because standard multivariable logistic regression is prone to sparse-data bias when non-events are limited (*n* = 14), a Firth's penalized likelihood multivariable logistic regression model was constructed (13). In accordance with the sample size constraints established in Section 2.3, the multivariable model was restricted to three covariates (Continuous GNRI, NLR ≥ 3, and Pathological Stage ≥ pT3). Odds ratios (OR) and 95% confidence intervals (CI) were calculated. Two-tailed *p*-values <0.05 were considered statistically significant.

**Table 1:** Preoperative and intraoperative patient characteristics stratified by preoperative GNRI threshold (GNRI < 94.5 vs. ≥ 94.5).

Variable	Low GNRI (< 94.5) (*n* = 31)	High GNRI (≥ 94.5) (*n* = 49)	Total Cohort (*N* = 80)	*p*-value
Demographic & Baseline Parameters				
Age (years), median (IQR)	70 (60–81)	68 (57–79)	69 (58–80)	0.384
Gender (Male/Female), *n* (%)	25 (80.6)/6 (19.4)	41 (83.7)/8 (16.3)	66 (82.5)/14 (17.5)	0.725
Smoking History, *n* (%)	23 (74.2)	36 (73.5)	59 (73.8)	0.942
Hypertension, *n* (%)	15 (48.4)	22 (44.9)	37 (46.3)	0.758
Diabetes Mellitus, *n* (%)	9 (29.0)	12 (24.5)	21 (26.3)	0.654
Cardiovascular Disease, *n* (%)	11 (35.5)	11 (22.4)	22 (27.5)	0.201
Body Mass Index (kg/m²), median (IQR)	23.8 (19.1–28.4)	25.9 (20.2–31.8)	25.0 (19.6–30.5)	**0.041** *
Body Mass Index < 25 kg/m², *n* (%)	20 (64.5)	20 (40.8)	40 (50.0)	**0.038** *
ASA Physical Status Score ≥ 3, *n* (%)	21 (67.7)	28 (57.1)	49 (61.3)	0.345
Charlson Comorbidity Index ≥ 5, *n* (%)	24 (77.4)	33 (67.3)	57 (71.3)	0.332
Nutritional & Laboratory Parameters				
Preoperative Serum Albumin (g/L), mean ± SD	32.4 ± 4.1	38.4 ± 4.3	36.1 ± 5.1	**< 0.001** *
C-Reactive Protein (mg/L), median (IQR)	8.2 (2.1–118.5)	3.8 (1.5–85.2)	4.5 (1.6–120.5)	**0.028** *
C-Reactive Protein ≥ 5 mg/L, *n* (%)	27 (87.1)	34 (69.4)	61 (76.3)	0.072
Neutrophil-to-Lymphocyte Ratio (NLR), median (IQR)	2.85 (1.10–4.20)	1.95 (0.75–3.10)	2.24 (0.84–3.64)	**0.019** *
NLR ≥ 3, *n* (%)	15 (48.4)	12 (24.5)	27 (33.8)	**0.028** *
Preoperative Hemoglobin (g/dL), median (IQR)	11.2 (8.8–13.8)	12.3 (9.4–15.1)	11.9 (9.1–14.7)	**0.035** *
Preoperative eGFR (mL/min/1.73 m²), mean ± SD	64.2 ± 25.1	68.5 ± 27.8	66.9 ± 26.8	0.482
Pathological & Intraoperative Data				
Postoperative Pathological Stage ≥ pT3, *n* (%)	23 (74.2)	31 (63.3)	54 (67.5)	0.312
Pathological Nodal Stage ≥ pN1, *n* (%)	13 (41.9)	17 (34.7)	30 (37.5)	0.521
Urinary Diversion (Ileal Conduit), *n* (%)	31 (100.0)	49 (100.0)	80 (100.0)	—
Neoadjuvant Chemotherapy, *n* (%)	6 (19.4)	10 (20.4)	16 (20.0)	0.910
Intraoperative Blood Loss (mL), median (IQR)	550 (350–750)	480 (300–650)	500 (300–700)	0.184
Intraoperative Blood Transfusion, *n* (%)	27 (87.1)	36 (73.5)	63 (78.8)	0.152
Operation Time (min), median (IQR)	330 (280–380)	315 (265–360)	320 (270–370)	0.275

SD, standard deviation; IQR, interquartile range; eGFR, estimated glomerular filtration rate; ASA, American Society of Anesthesiologists; NLR, neutrophil-to-lymphocyte ratio. Bold values indicate statistically significant *p*-values (*p* < 0.05).

* Statistically significant (*p* < 0.05).

**Table 2:** 90-day postoperative outcomes, hospital/ICU stay, and complication subtypes stratified by preoperative GNRI cutoff (GNRI < 94.5 vs. ≥ 94.5).

Postoperative Parameter/Outcome	Low GNRI (< 94.5) (*n* = 31)	High GNRI (≥ 94.5) (*n* = 49)	Total Cohort (*N* = 80)	*p*-value	Odds Ratio (95% CI)
Hospital Length of Stay (days), median (IQR)	11 (7–16)	8 (5–11)	9 (5–13)	**0** **.** **008** *	—
Hospital Length of Stay > 8 days, *n* (%)	23 (74.2)	21 (42.9)	44 (55.0)	**0**.006*	3.83 (1.42–10.33)
Postoperative ICU Stay (days), median (range)	2 (1–28)	1 (1–14)	2 (1–28)	**0**.021*	—
Postoperative ICU Stay > 1 day, *n* (%)	21 (67.7)	19 (38.8)	40 (50.0)	**0**.012*	3.32 (1.28–8.58)
Overall Complications (CD Grade I–V), *n* (%)	28 (90.3)	38 (77.6)	66 (82.5)	0.142	2.70 (0.69–10.55)
Minor Complications (CD Grade I–II), *n* (%)	13 (41.9)	17 (34.7)	30 (37.5)	0.521	1.36 (0.54–3.43)
Major Complications (CD Grade III–IV), *n* (%)	12 (38.7)	19 (38.8)	31 (38.8)	0.995	0.99 (0.39–2.51)
Infectious Complications, *n* (%)	21 (67.7)	17 (34.7)	38 (47.5)	**0**.004*	3.95 (1.51–10.32)
Gastrointestinal Complications, *n* (%)	23 (74.2)	18 (36.7)	41 (51.3)	**0**.001*	4.95 (1.84–13.31)
Cardiovascular Complications, *n* (%)	8 (25.8)	2 (4.1)	10 (12.5)	**0**.005*	8.17 (1.60–41.72)
Wound Healing Deficiency, *n* (%)	12 (38.7)	13 (26.5)	25 (31.3)	0.258	1.75 (0.66–4.62)
Urinary Complications, *n* (%)	8 (25.8)	8 (16.3)	16 (20.0)	0.301	1.78 (0.58–5.46)
Postoperative TPN Requirement, *n* (%)	27 (87.1)	35 (71.4)	62 (77.5)	0.108	2.70 (0.78–9.32)
ICU Vasopressor Support, *n* (%)	8 (25.8)	7 (14.3)	15 (18.8)	0.201	2.08 (0.67–6.50)
90-Day Mortality (CD Grade V), *n* (%)	3 (9.7)	2 (4.1)	5 (6.3)	0.368	2.52 (0.39–16.14)

CD, Clavien-Dindo; ICU, intensive care unit; TPN, total parenteral nutrition; CI, confidence interval. Bold values indicate statistically significant *p*-values (*p* < 0.05).

* Statistically significant (*p* < 0.05).

## 3 Results

The study included 80 consecutive patients undergoing open radical cystectomy. The median patient age was 69 years (IQR: 58–80), and 82.5% (*n* = 66) were male. All 80 patients (100.0%) underwent ileal conduit urinary diversion. High comorbidity burden was evident: 61.3% presented an ASA score ≥3, and 71.3% had a Charlson Comorbidity Index ≥5. Mean preoperative GNRI score for the overall cohort was 94.81 ± 7.24 (range: 78.4–106.2). Preoperative demographic, clinical, laboratory, and intraoperative characteristics stratified by the ROC-derived GNRI cutoff (< 94.5 vs. ≥ 94.5) are summarized in Table 1. Patients in the Low GNRI cohort exhibited significantly lower mean serum albumin (32.4 ± 4.1 vs. 38.4 ± 4.3 g/L, *p* < 0.001) and median BMI (23.8 vs. 25.9 kg/m², *p* = 0.041) compared to those in the High GNRI group. Additionally, Low GNRI status was characterized by heightened baseline inflammation, evidenced by significantly higher C-reactive protein (median 8.2 vs. 3.8 mg/L, *p* = 0.028) and neutrophil-to-lymphocyte ratios (median 2.85 vs. 1.95, *p* = 0.019; NLR ≥ 3: 48.4% vs. 24.5%, *p* = 0.028), alongside lower baseline hemoglobin levels (median 11.2 vs. 12.3 g/dL, *p* = 0.035). Age, gender, comorbidity scores, pathological tumor stage, and intraoperative variables were comparable between the two groups. Detailed breakdown of neoadjuvant and adjuvant chemotherapy protocols is provided in Supplementary Table S1.

Within 90 days of surgery, 66 patients (82.5%) experienced at least one complication (minor CD I–II: 37.5%, *n* = 30; major CD III–IV: 38.8%, *n* = 31; mortality CD V: 6.3%, *n* = 5). In Receiver Operating Characteristic (ROC) curve analysis, continuous preoperative GNRI yielded an Area Under the Curve (AUC) of 0.628 (95% CI: 0.485–0.771) for predicting overall 90-day postoperative complications. The optimal cohort-specific threshold was derived via Youden's Index at GNRI = 94.5 (Sensitivity: 71.2%, Specificity: 57.1%) (Figure 1).

**Figure 1:**
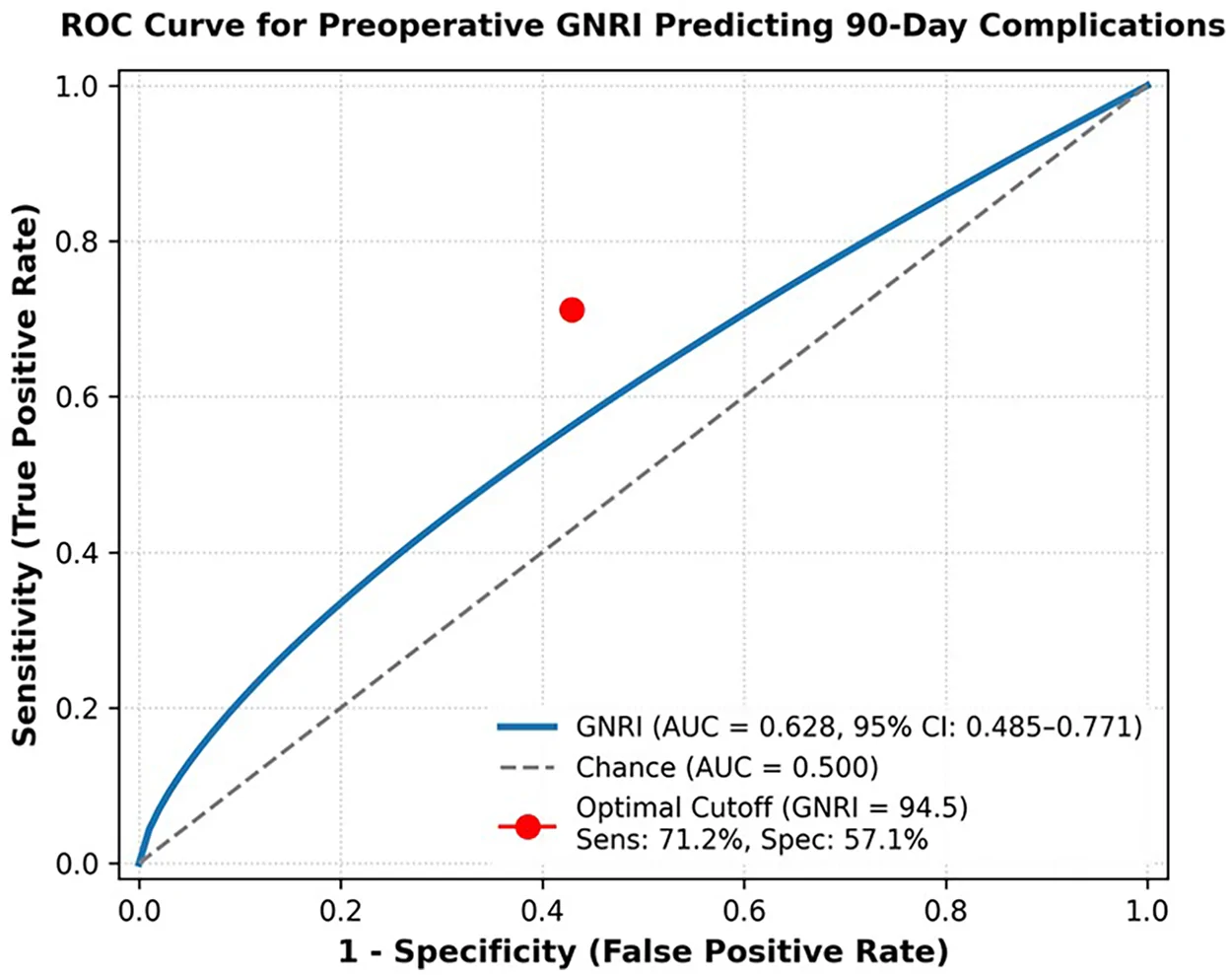
Receiver operating characteristic (ROC) curve analysis of preoperative geriatric nutritional risk index (GNRI) for predicting 90-day overall postoperative complications following open radical cystectomy. Area Under the Curve (AUC) = 0.628 (95% CI: 0.485–0.771). The red marker denotes the optimal cohort-specific cutoff derived via Youden's Index (GNRI = 94.5; Sensitivity: 71.2%, Specificity: 57.1%).

Stratification by the empirical cutoff (GNRI < 94.5 vs. ≥ 94.5) revealed that while overall complication rates did not differ significantly (90.3% vs. 77.6%, *p* = 0.142), patients with low GNRI experienced striking, statistically significant elevations in specific severe morbidity subtypes (Table 2):

- Infectious Complications: 67.7% (21/31) in GNRI < 94.5 vs. 34.7% (17/49) in GNRI ≥ 94.5 (OR: 3.95, 95% CI: 1.51–10.32, *p* = 0.004).
- Gastrointestinal Complications: 74.2% (23/31) in GNRI < 94.5 vs. 36.7% (18/49) in GNRI ≥ 94.5 (OR: 4.95, 95% CI: 1.84–13.31, *p* = 0.001).
- Cardiovascular Adverse Events: 25.8% (8/31) in GNRI < 94.5 vs. 4.1% (2/49) in GNRI ≥ 94.5 (OR: 8.17, 95% CI: 1.60–41.72, *p* = 0.005).

When evaluating historical cutoffs, GNRI < 92 was associated with gastrointestinal complications (76.5% vs. 44.4%, *p* = 0.016) and cardiovascular events (29.4% vs. 7.9%, *p* = 0.028), whereas GNRI < 98 significantly correlated with infectious complications (58.3% vs. 31.3%, *p* = 0.021).

In univariable logistic regression, elevated NLR (≥3, OR: 8.475, *p* = 0.046), intraoperative blood transfusion (OR: 3.745, *p* = 0.037), and advanced pathological stage (≥pT3, OR: 5.181, *p* = 0.008) were significantly associated with overall 90-day complications. Continuous GNRI demonstrated a risk trend per 1-unit decrease (OR: 1.076, 95% CI: 0.977–1.185, *p* = 0.138). In the 3-covariate Firth's multivariable model, advanced pathological tumor stage (≥pT3) was confirmed as the sole independent predictor of overall 90-day complications (OR: 4.187, 95% CI: 1.212–14.463, *p* = 0.024) (Table 3).

**Table 3:** Univariable and Firth's multivariable logistic regression analysis of predictors for overall 90-day postoperative complications.

Clinical Factor/Parameter	No Complication (*n* = 14)	Complications (*n* = 66)	Univariable OR (95% CI)	Univariable *p*-value	Multivariable OR (95% CI)*	Multivariable p-value*
Age (continuous, years)	67.2 ± 8.1	68.9 ± 7.6	1.027 (0.957–1.102)	0.461	—	—
Gender (Female vs. Male)	2 (14.3%)	12 (18.2%)	1.333 (0.263–6.753)	0.728	—	—
Body Mass Index (< 25 kg/m²)	6 (42.8%)	34 (51.5%)	1.417 (0.443–4.534)	0.557	—	—
GNRI (Continuous, per 1-unit decrease)	97.42 ± 5.81	94.25 ± 7.42	1.076 (0.977–1.185)	0.138	1.052 (0.948–1.168)	0.342
GNRI Cutoff < 94.5 (ROC-derived)	3 (21.4%)	28 (42.4%)	2.702 (0.692–10.551)	0.152	—	—
Preoperative Albumin (< 35 g/L)	2 (14.3%)	28 (42.4%)	4.421 (0.916–21.347)	0.064	—	—
NLR ≥ 3	1 (7.1%)	26 (39.4%)	8.475 (1.042–66.667)	0.046*	4.215 (0.684–25.981)	0.122
CRP ≥ 5 mg/L	9 (64.3%)	52 (78.8%)	2.062 (0.596–7.143)	0.253	—	—
ASA Physical Status ≥ 3	8 (57.2%)	41 (62.1%)	1.230 (0.382–3.968)	0.729	—	—
Intraoperative Blood Transfusion	8 (57.2%)	55 (83.3%)	3.745 (1.085–12.987)	0.037*	2.412 (0.618–9.412)	0.204
Pathological Tumor Stage ≥ pT3	5 (35.7%)	49 (74.2%)	5.181 (1.524–17.544)	0.008*	4.187 (1.212–14.463)	0.024*

*Multivariable model constrained to 3 covariates based on Riley's pmsampsize methodology given 14 non-events, using Firth's penalized likelihood logistic regression. Statistically significant (*p* < 0.05). Bold values indicate statistically significant *p*-values (*p* < 0.05).

## 4 Discussion

Open radical cystectomy with pelvic lymphadenectomy and urinary diversion remains a formidable surgical procedure with significant perioperative morbidity. In this study of 80 patients, we rigorously evaluated the prognostic capability of preoperative GNRI. Our principal findings indicate that while preoperative GNRI—analyzed either as a continuous variable or via an empirical ROC threshold (GNRI < 94.5)—does not serve as an independent predictor of overall 90-day complications, lower GNRI scores are strongly and specifically associated with heightened risks of infectious, gastrointestinal, and cardiovascular adverse events.

Historically, the thresholds of 92 and 98 were defined by Bouillanne et al. (8) to stratify moderate/severe and mild nutritional risk in elderly hospitalized patients. In surgical oncology cohorts, these thresholds have been widely cited (9, 10). However, arbitrary categorization of continuous biological parameters can result in substantial statistical power loss. By analyzing GNRI continuously and deriving a cohort-specific ROC cutoff (GNRI = 94.5), we demonstrated that overall surgical morbidity in open radical cystectomy is overwhelmingly driven by locally advanced tumor stage (≥pT3, OR: 4.187), rather than baseline nutritional status alone. This finding contrasts with previous reports in laparoscopic or robotic cystectomy series as well as geriatric trauma populations (9, 10). This discrepancy can be attributed to the profound physiological stress inherent to open radical cystectomy. Open RC involves extensive tissue trauma, prolonged operative duration, substantial fluid shifts, and direct intestinal manipulation, eliciting a profound systemic inflammatory surge. This severe surgical stress exerts a dominant impact on postoperative recovery, effectively overshadowing mild-to-moderate baseline nutritional variations. In contrast, minimally invasive approaches entail significantly reduced surgical trauma, allowing underlying nutritional frailty to manifest more prominently as an independent prognostic driver. Additionally, an inherent mathematical limitation of the GNRI formula is the capping of the current-to-ideal body weight ratio at 1.0 for overweight and obese individuals. This restriction prevents the index from capturing the heightened perioperative risk profiles associated with obesity, which may further account for its lack of independent prognostic power in overall complications.

A critical finding needing thorough clarification is the strong association between low GNRI and gastrointestinal complications (74.2% vs. 36.7%, OR: 4.95, *p* = 0.001). As queried by reviewers, this association is intimately linked to the urinary diversion technique employed. In our cohort, 100.0% of patients underwent ileal conduit urinary diversion, which necessitates distal ileal resection and hand-sewn or stapled bowel re-anastomosis. Malnourished patients with low GNRI present with chronic hypoalbuminemia, impaired collagen synthesis, and microvascular compromise. Following extensive surgical dissection, bowel wall edema and delayed mucosal healing predispose these patients to prolonged paralytic ileus, mechanical bowel obstruction, and anastomotic leakage. Thus, low GNRI acts as a direct surrogate for impaired bowel recovery in ileal conduit surgery.

Our findings align with and expand upon recent literature emphasizing perioperative nutrition and dietary habits in bladder cancer. As highlighted by Buonerba et al. (14), dietary patterns, nutraceuticals, and targeted nutritional supplements play a crucial role in modulating systemic inflammation, gut microbiota, and immune competence in bladder cancer patients undergoing multimodal therapy. Integrating objective screening tools like GNRI into prehabilitation programs enables surgical teams to identify vulnerable patients who would benefit from specialized enteral immunonutrition prior to cystectomy.

Furthermore, personalized risk prediction models and nomograms have gained significant traction in urological oncology. Outstanding examples include the CRAB nomogram developed by Cicione et al. (15) and the validated COBRA nomogram evaluated by De Nunzio et al. (16) for predicting survival outcomes after radical cystectomy. While these nomograms primarily incorporate pathological and clinical variables, incorporating objective nutritional metrics like GNRI alongside systemic inflammatory biomarkers could further refine their predictive accuracy for short-term perioperative morbidity.

In addition, the prognostic significance of preoperative inflammatory markers and histopathological parameters in urothelial malignancies has been thoroughly established. Mastroianni et al. (17) highlighted the critical prognostic role of histopathological features such as detrusor muscle presence in urothelial carcinoma, while Antonucci et al. (18) demonstrated the utility of preoperative Neutrophil-to-Lymphocyte Ratio (NLR) as an objective prognostic tool in upper tract urothelial carcinoma. In our cohort, NLR ≥3 was significantly associated with overall complications in univariable analysis (OR: 8.475, *p* = 0.046), reinforcing the synergistic impact of systemic inflammation and nutritional depletion on surgical outcomes.

From a clinical management standpoint, our study underscores that GNRI should not be viewed as an isolated, blanket predictor of overall surgical risk, but rather as an actionable component of Enhanced Recovery After Surgery (ERAS) protocols. Preoperative identification of low GNRI (<94.5) allows clinicians to initiate targeted prehabilitation—including oral immunonutrition supplements, pre-cystectomy physical conditioning, and intensified postoperative surveillance for ileus and sepsis.

Several limitations of this study warrant consideration. First, its retrospective, single-center design introduces inherent selection bias. Second, the modest sample size (*N* = 80) and small number of non-events (*n* = 14) restricted multivariable modeling; however, overfitting was strictly mitigated in the 3-covariate Firth's multivariable model. Third, serum albumin—a core constituent of GNRI—can be influenced by tumor-induced systemic inflammation, making it difficult to completely separate pure malnutrition from cancer cachexia. Multi-center prospective studies with larger cohorts are needed to validate these findings.

## 5 Conclusions

In conclusion, preoperative GNRI is not an independent predictor of overall 90-day postoperative complications following open radical cystectomy with ileal conduit; overall morbidity in this setting is primarily driven by advanced pathological stage (≥pT3). However, lower preoperative GNRI scores (<94.5) are significantly associated with higher rates of specific infectious, gastrointestinal, and cardiovascular adverse events. Therefore, GNRI serves as an objective, practical screening instrument within ERAS pathways to guide targeted prehabilitation and perioperative nutritional optimization prior to major radical surgery.

## Data Availability

The original contributions presented in the study are included in the article/Supplementary Material, further inquiries can be directed to the corresponding author.

## Supplementary material

SUPPLEMENTARY TABLE S1 Detailed breakdown of neoadjuvant and adjuvant chemotherapy protocols in the study cohort.

## References

[1] Witjes JA Bruins HM Cathomas R Compérat EM Cowan NC Gakis G. EAU guidelines on muscle-invasive and metastatic bladder cancer: summary of the 2020 guidelines. Eur Urol. (2021) 79(1):82–104. 10.1016/j.eururo.2020.03.055 32360052

[2] Witjes JA Bruins HM Cathomas R Compérat EM Cowan NC Gakis G. Muscle invasive and metastatic bladder cancer: EAU guidelines. Eur Urol. (2024) 85(3):210–25. 10.1016/j.eururo.2023.08.016

[3] Shabsigh A Korets R Vora KC Brooks CM Cronin AM Savage C. Defining early morbidity of radical cystectomy for bladder cancer using a standardized reporting methodology. J Urol. (2009) 181(4):1642–7. 10.1016/j.eururo.2008.07.031 18675501

[4] Shabsigh A Korets R Vora KC Brooks CM Cronin AM Savage C. Defining early morbidity of radical cystectomy for patients with bladder cancer using a standardized reporting methodology. Eur Urol. (2009) 55(1):164–74. 10.1016/j.eururo.2008.07.031 18675501

[5] Psutka SP Boorjian SA Moynagh MR Schmit GD Frank I Carrasco A. Mortality after radical cystectomy: impact of obesity versus adiposity after adjusting for skeletal muscle wasting. J Urol. (2015) 193(5):1507–13. 10.1016/j.juro.2014.11.088 25464002 PMC4556235

[6] Rink M Sharifi N Fritsche HM Aziz A Miller F Kluth LA. Impact of preoperative anemia on oncologic outcomes of upper tract urothelial carcinoma treated with radical nephroureterectomy. J Urol. (2014) 191(2):316–22. 10.1016/j.juro.2013.09.010 24036235

[7] Gregg JR Cookson MS Phillips S Salem S Chang SS Clark PE. Effect of preoperative nutritional deficiency on mortality after radical cystectomy for bladder cancer*.* J Urol*.* (2011) 185(1):90–6. 10.1016/j.juro.2010.09.021 21074802 PMC3049248

[8] Bouillanne O Morineau G Dupont C Coulombel I Vincent J-P Nicolis I. Geriatric nutritional risk index: a new tool for evaluating nutritional status in elderly medical patients. Am J Clin Nutr. (2005) 82(4):777–83. 10.1093/ajcn/82.4.777 16210706

[9] Riveros C Jazayeri SB Chalfant V Ahmed F Bandyk M Balaji KC. The geriatric nutritional risk index predicts postoperative outcomes in bladder cancer: a propensity score-matched analysis. J Urol. (2022) 207(4):797–804. 10.1097/JU.0000000000002342 34854753

[10] Fukuta K Daizumoto K Sasaki Y Izumi K Kadoriku F Utsunomiya S. Geriatric nutritional risk index predicts postoperative complications in elderly patients undergoing robot-assisted radical cystectomy. Int J Urol. (2025) 32(8):944–50. 10.1111/iju.70070 40269442

[11] Riley RD Snell KIE Ensor J Burke DL Harrell Jr FE Moons KGM. Minimum sample size for developing a multivariable prediction model: pART II—binary and time-to-event outcomes. Stat Med. (2019) 38(7):1276–96. 10.1002/sim.7992 30357870 PMC6519266

[12] Clavien PA Barkun J De Oliveira ML Vauthey JN Dindo D Schulick RD. The Clavien-Dindo classification of surgical complications: five-year experience. Ann Surg. (2009) 250(2):187–96. 10.1097/SLA.0b013e3181b13ca2 19638912

[13] Firth D. Bias reduction of maximum likelihood estimates. Biometrika. (1993) 80(1):27–38. 10.1093/biomet/80.1.27

[14] Buonerba C Ingenito C Di Trolio R Cappuccio F Rubino R Piscosquito A. Unraveling the dietary puzzle: exploring the influence of diet, nutraceuticals, and supplements on bladder cancer risk, outcomes, and immunotherapy efficacy: insights from the BLOSSOM study and beyond. Oncol Ther. (2024) 12(2):189–95. 10.1007/s40487-024-00266-7 38416326 PMC11187017

[15] Cicione A Simone G Lombardo R Franco A Nacchia A Ghezzo N. Development of a pocket nomogram to predict cancer and disease specific survival after radical cystectomy for bladder cancer: the CRAB nomogram. Clin Genitourin Cancer. (2023) 21(1):108–14. 10.1016/j.clgc.2022.08.011 36175311

[16] De Nunzio C Franco A Simone G Tuderti G Anceschi U Brassetti A. Validation of the COBRA nomogram for the prediction of cancer specific survival in patients treated with radical cystectomy for bladder cancer: an international wide cohort study. Eur J Surg Oncol. (2021) 47(10):2646–50. 10.1016/j.ejso.2021.04.035 33966945

[17] Mastroianni R Brassetti A Krajewski W Zdrojowy R Salhi YA Anceschi U. Assessing the impact of the absence of detrusor muscle in ta low-grade urothelial carcinoma of the bladder on recurrence-free survival. Eur Urol Focus. (2021) 7(6):1324–31. 10.1016/j.euf.2020.08.007 32900676

[18] Antonucci M Defidio L De Dominicis M Russo N Nacchia A Lombardo R. Utility of preoperative neutrophil/lymphocyte ratio as a new objective prognostic tool in endoscopically treated upper tract urothelial carcinoma: a retrospective evaluation. J Endourol. (2020) 34(9):993–1000. 10.1089/end.2020.0283 32600070

