# Supplementary material for: Association between the preoperative geriatric nutritional risk index and 90-day complications after open radical cystectomy: a retrospective cohort study
Source: Front Surg. 2026 Oct 29;13:1987253. doi: 10.3389/fsurg.2026.1987253 (PMC13755301; doi:10.3389/fsurg.2026.1987253)
Supplement: Supplementary file 1 [file Table1.docx]

Supplementary Table S1. Detailed Breakdown of Neoadjuvant and Adjuvant Chemotherapy Regimens

Overview of perioperative systemic treatment protocols (N = 80)

Chemotherapy Protocol	Neoadjuvant CT (n = 16), n (%)	Adjuvant CT (n = 25), n (%)
Gemcitabine + Cisplatin (GC)	12 (75.0)	11 (44.0)
Gemcitabine + Carboplatin (GemCarbo)	2 (12.5)	10 (40.0)
Enfortimab Vedotin + Pembrolizumab (EVP)	1 (6.3)	0 (0.0)
dose-dense MVAC (ddMVAC)	1 (6.3)	0 (0.0)
Pembrolizumab Monotherapy	0 (0.0)	4 (16.0)
